# Barriers to training in laparoscopic surgery in low- and middle-income countries: A systematic review

**DOI:** 10.1177/0049475521998186

**Published:** 2021-04-13

**Authors:** Ellen Wilkinson, Noel Aruparayil, J Gnanaraj, Julia Brown, David Jayne

**Affiliations:** 1 Nuffield Centre for International Health and Development, 4468 University of Leeds, Leeds, UK; 2 Leeds Institute of Medical Research at St. James’s, 4468 University of Leeds, Leeds, UK; 3 Rural Surgery Research and Training Center, Shanthi Bhavan Medical Center, Biru, India; 4 Leeds Institute of Clinical Trials Research, 4468 University of Leeds, Leeds, UK

**Keywords:** Laparoscopy, training, LMIC

## Abstract

Laparoscopic surgery has the potential to improve care in resource-deprived low- and-middle-income countries (LMICs). This study aims to analyse the barriers to training in laparoscopic surgery in LMICs. Medline, Embase, Global Health and Web of Science were searched using ‘LMIC’, ‘Laparoscopy’ and ‘Training’. Two researchers screened results with mutual agreement. Included papers were in English, focused on abdominal laparoscopy and training in LMICs. PRISMA guidelines were followed; 2992 records were screened, and 86 full-text articles reviewed to give 26 key papers. Thematic grouping identified seven key barriers: *funding*; availability and maintenance of *equipment*; local access to experienced laparoscopic *trainers*; *stakeholder dynamics*; lack of knowledge on effective *training curricula*; *surgical departmental structure* and *practical opportunities* for trainees. In low-resource settings, technological advances may offer low-cost solutions in the successful implementation of laparoscopic training and improve access to surgical care.

## Introduction

Five billion people worldwide lack access to safe, affordable surgical care.^1^ This problem is particularly acute in low- and lower middle-income countries, where it applies to nine out of ten people.^1^ As the global burden of disease moves from communicable to non-communicable diseases, evidence has shown the strong impact that access to essential surgery can have.^2^

Laparoscopic surgery is increasingly used in high-income countries (HICs)^3^ because of the benefits for patients and healthcare systems.^4^ In comparison to open surgery, laparoscopy is associated with reduced infection rates,^5^ which is particularly problematic in low-income countries (LICs)^6^ where rates are up to 25 times greater than in HICs.^5^ Laparoscopy helps to reduce postoperative pain^7^ and shortens hospital stay,^8^ which benefits LOW AND MIDDLE INCOME COUNTRIES (LMICs) where hospital beds are scarce, and families often rely on a single breadwinner.

Previous studies have suggested that training in laparoscopic surgery in low-resource settings is feasible,^9^ but the uptake has been slow due to various barriers,^10,11^ and cost-effectiveness is debated.^3^

This study aimed to systematically review the literature to identify barriers to a sustainable implementation of laparoscopic surgical training for abdominal conditions and suggest potential solutions.

## Methods

A protocol for this review is available on PROSPERO at https://www.crd.york.ac.uk/prospero/display_record.php?ID=CRD42019124535

The following databases were searched in April 2020:

- Ovid MEDLINE(R) and Epub Ahead of Print, In-Process & Other Non-Indexed Citations and Daily (1946 to 3 April 2020)
- Embase (1974 to 3 April 2020)
- Global Health (1973 to 2020 Week 15).

Web of Science (1900–2020) was also searched, including the following databases:

- Web of Science Core Collection
- Book Citation Index
- BIOSIS
- Current Contents Connect
- Data Citation Index
- Korean Journal Database
- Russian Science Citation Index
- SciELO.

Searches were based on the key terms ‘LMIC’, ‘Laparoscopy’ and ‘Training’. Synonyms, truncation and Boolean operators were used to produce a thorough search strategy, with search terms mapped to relevant subject headings in each database, and ‘exploded’ where appropriate. See Appendix 1 for the full search strategy.

Results were filtered to be English language only, and other inclusion/exclusion criteria were applied during manual review (see Appendix 2). Abdominal laparoscopy was the focus of this review because acute abdominal conditions are a significant cause of premature mortality in many LMICs.^12^ Papers on advanced laparoscopic surgeries, such as transplants or robotics, were excluded because facilities undertaking such procedures have likely overcome any barriers. Citation searching was used to find additional relevant papers.

Two authors independently screened results by title and abstract, undertook full-text review and discussed results to reach concordance.

Data extraction and narrative synthesis of barriers were performed by one author (EW) using Microsoft Excel and reviewed by a co-author (NA).

## Results

**Figure 1.**
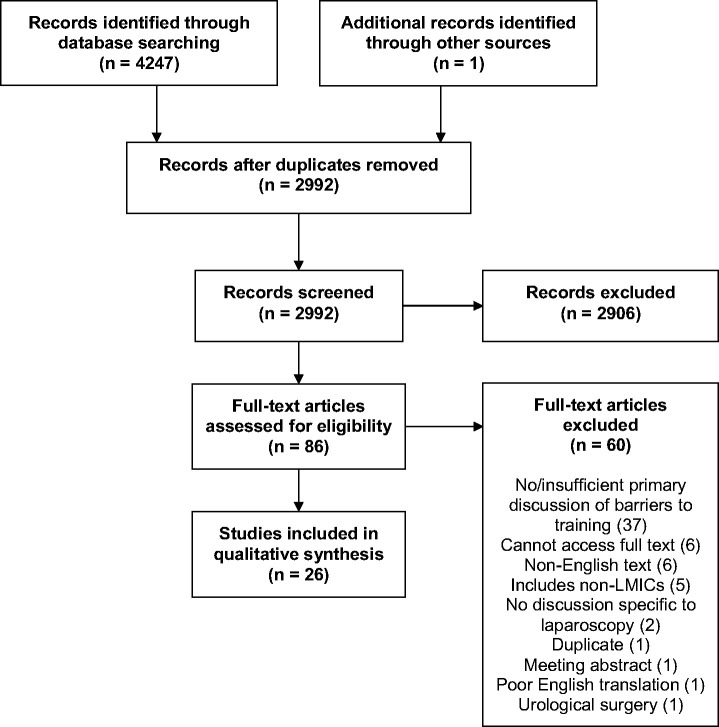
Flow diagram summarising search and screening strategy, based on PRISMA flow diagram^13^.

Figure 1 shows the PRISMA flow diagram, detailing the search and screening strategy.

Twenty-six papers were included, covering data from at least 18 LMICs. Table 1 shows the papers included and the barriers to laparoscopic surgery identified.

**Table 1.** Summary of included studies, their geographical distribution and the seven key barriers found.

Country	Ref	Year	First author	Funding	Equipment	Trainers	Stakeholder dynamics	Surgical department structure	Training structure/ curriculum	Opportunity to practise
LMICs	9	2017	Alfa-Wali		✓	✓		✓	✓	
Africa										
Botswana	28	2009	Okrainec		✓				✓	
	17	2010	Okrainec	✓	✓				✓	
	11	2015	Bedada		✓		✓	✓	✓	✓
Ethiopia	29	2016	Morrow		✓	✓			✓	✓
Ghana	19	2014	Andreatta	✓	✓					✓
Kenya	24	2014	Long			✓				✓
Madagascar	14	2018	Ghesquiere	✓	✓				✓	✓
Nigeria	4	2011	Afuwape	✓	✓	✓	✓	✓		
	20	2014	Ekwunife	✓			✓		✓	
Rwanda	35	2018	Robertson		✓	✓		✓	✓	✓
South Africa	16	2007	Apostolou	✓	✓		✓		✓	✓
	25	2018	Patel		✓	✓				
Tanzania	23	2014	Beard	✓	✓				✓	✓
West African country	7	2013	Choy				✓	✓		
Asia										
China	18	2007	Li		✓		✓		✓	✓
India	32	2005	Supe	✓	✓				✓	
	21	2009	Supe						✓	✓
Mongolia	27	2011	Straub*		✓	✓	✓			✓
	15	2012	Vargas*		✓	✓	✓	✓	✓	
Pakistan	34	2014	Jan	✓	✓	✓	✓		✓	✓
	26	2019	Nofal		✓		✓			✓
Central America										
Haiti	31	2020	Harvey		✓					
Nicaragua and Bolivia	28	1996	Asbun		✓	✓	✓		✓	✓
South America										
Bolivia	*See Asbun, 1996*									
Brazil	33	2015	Loureiro	✓	✓		✓	✓	✓	
	22	2016	Fernandes	✓	✓		✓		✓	✓

*Address the same training programme.

More than half of the included papers focused on African countries, with data collected between 2007 and 2018. Seven papers looked at Asia between 2005 and 2019, and further papers were included from the Americas. Sixteen of the papers described the implementation of laparoscopic training programmes, and most of these were small scale; all but two involved 20 or fewer participants and lasted only a few days. The most common barriers described were equipment (22 papers) and training structure and curriculum (18 papers).

### Funding

Establishing or attending a training course often requires substantial investment from the local hospital, Ministry of Health^11^ or international partners.^14^ A successful laparoscopy training programme requires funding for a variety of staff, including nurses^11^ and support staff to maintain equipment.^15^

Surgeons may choose to self-fund attendance at an established course, which are often in bigger cities or overseas, meaning the cost of travel and attendance is often prohibitive.^16,17^ If hospitals fund a surgeon’s travel, this is usually reserved for the most senior staff.^18^

If the costs of establishing a local training programme are feasible, the costs to sustain it can be limiting.^19^ European stakeholders established a five-day laparoscopy training workshop in Nigeria, but financial constraints prevented plans for its periodic repetition and sustainability.^20^

Several alternatives to live training on humans have been described, including virtual reality,^21^ cadavers^22^ and animal models,^14^ but these can be expensive to establish and maintain.^21,22^ Beard et al. developed a low-cost box trainer for use by 14 surgeons in Tanzania, with supplies sourced locally for under US$100.^23^ The assessment scores of all participants improved significantly after training.^23^ Long et al. developed a ‘low-cost’ curriculum in Kenya, costing only US$50, but this only included consumables and relied upon box trainers already available.^24^Neither of the Beard nor Long studies included the cost of computers or laparoscopic instruments, which were donated by the trainers.^23,24^

### Equipment

Many studies have highlighted the lack of equipment or resources as one of the most significant barriers to laparoscopic training.^16,22,25,26^ Poor financing of public hospitals and ‘unrealistic’ pricing of laparoscopic equipment are cited.^16^

Most programmes implementing laparoscopic training relied upon equipment donations from international stakeholders.^14,15,31,17,19,23,24,27–30^ Even then, equipment constraints often limited the number of participants and the ability to complete training,^19,29^ which probably contributed to low assessment scores.^30^

Lack of equipment often means laparoscopic training programmes are the privilege of only the larger hospitals and cities in LMICs.^18^ A Mumbai training programme used cadavers shared between trainees, but obtaining donated or unclaimed cadavers may be difficult.^31^ In Brazil, most residencies are conducted in public hospitals, where there is a paucity of laparoscopic equipment,^32^ so surgeons in smaller hospitals, therefore, need to wait until later in their career for laparoscopic training.^18^

Various measures to reduce constraints due to lack of equipment have been described.^11,28^ Decontamination and reuse of disposable instruments led to equipment failure after prolonged use,^28^ highlighting the importance of replenishment strategies.^28,29^ Equipment is often bought that is outdated and can deteriorate quickly,^32^ particularly when there is poor maintenance.^28,33^

Insufficient transport systems, especially in rural areas, can limit the delivery of equipment and other resources.^27^ Pressures on time means that any delays in obtaining equipment hinder the effectiveness of training.^27^

### Trainers

The lack of experienced trainers in more remote LMIC regions^9,34^ is a major barrier to laparoscopic training and utilisation.^25,35^ In Nigeria, training programmes are often run by volunteers from HICs, but these opportunities are rare and require volunteers accustomed to teaching in alien, resource-limited environments.^4^

Eleven papers described multiple barriers in training programmes that relied solely on expatriate surgeons as expert trainers.^11,14,31,15,19,20,23,24,27,28,30^ Travel and time constraints of American trainers limited courses in Bolivia and Nicaragua to a maximum of five days.^28^ Short courses showed mixed results^20,29,30^ and have been criticised, compared to those spanning several weeks, for limiting repetition and accumulation of knowledge.^17,30,32,34^ The language barrier between American trainers and Mongolian trainees on a laparoscopic cholecystectomy programme was a significant setback^27^ with a lack of medically skilled translators necessitating unplanned collaboration with bilingual native surgeons.^15^

To circumvent the inconvenience and expense of travel for foreign trainers, strategies for remote training have been tried. Surgeons from Canada conducted tele-proctoring for eight surgeons from Botswana, whilst a control group carried out self-practice using an instructional DVD.^17^ The tele-proctoring group had significantly higher scores in the post-training test than the self-practice group.^17^ However, the contributing factors to success were unclear and whether this was due to the expert feedback, the increased practise time compared to the self-practice group, or simply the routine of weekly training sessions.^17^ Frequent internet connectivity issues and local power outages can limit the effectiveness of tele-proctoring, even when performed in urban areas.^17^

### Stakeholder dynamics

Laparoscopic skills are often taught to the most senior surgeons, who are expected to pass them on to the rest of the team.^7^ However, the attraction of working environments in HICs means that retention of medical personnel is often problematic, particularly in Africa.^4^ For those returning to their native countries, conservative attitudes to surgical advances^20,27,32^ and disinterest from older surgeons^4,7^often means that learnt skills are not implemented.^7^ Lack of interest from the preceptor and lack of encouragement or confidence from seniors have been identified as training barriers by residents in Brazil, Pakistan and South Africa.^16,22,26^ Deep societal respect for age and experience in some cultures means that residents are unlikely to push their seniors for training opportunities.^7^

Dynamics between local and foreign stakeholders can also be a barrier to effective training programmes. In Botswana, rumours developed that a training programme was designed primarily for the research interests of the external partners, rather than for the benefit of the trainees.^11^ Widely publicised complications after laparoscopic cholecystectomy in Mongolia led to public distrust, and local government officials questioned the safety and sustainability of a laparoscopic training course,^27^ which seemed to discourage patients, with many favouring laparotomy.^27^

### Surgical department structure

Laparoscopy is well-suited to subspecialisation as it allows individual development of highly technical skills.^7^ There is limited capacity for this in rural settings of LMICs due to the workforce constraints, requiring surgeons to be proficient in a wide range of procedures.^9^

Poor departmental organisation and processes can add further barriers. In a Mongolian training programme, turnover of operating rooms was slow due to delays in equipment sterilisation.^15^ To prevent poor practice under time-pressure and increased infection risk, the number of training operations was reduced.^15^ Lack of human resources, a common issue in LMICs, may also have contributed.^15^

### Training structure and curriculum

A lack of validated curricula, assessment tools and unclear training objectives leads to widespread variation in resident experience,^22^ which may contribute to the early demise of training courses.^32^

The surgical mantra of ‘see one, do one, teach one’ still exists in many LMICs,^9^ leading to unstructured skills acquisition and possibly unsafe practices.^9^ The traditional apprentice/mentor model of surgical training has been shown to be highly subjective.^14^

Supervised practice in the operating room for inexperienced trainees, as is common for open surgery, is less effective for laparoscopy.^21^ The greater risk of complications means it is ethically questionable,^21^ particularly when laparoscopic simulation methods are available.

Training curricula need to be designed with consideration of local needs.^24^ Nurses who attended a training course in South Africa reported a basic experience that did not meet their expectations.^11^ Often, foreign experts use training guidelines from their own country,^28,33^ which are not realistic or effective in resource-limited settings.^28^

### Opportunity to practise

Multiple papers suggest lack of opportunity for skills development in the operating room.^14,16,23,24,28,29^ South African surgeons estimated that 24 laparoscopic cholecystectomies should be performed to achieve competency, whereas only 19.2 could be completed by the end of their training programme.^16^

Contributing factors include theatre time constraints,^16^ lack of proctoring and supervision,^28^ lack of staff trained in managing a laparoscopic theatre^14,28^ and limited laparoscopic case volume^22,29^ – especially in smaller hospitals.^18^ Ethiopian surgeons were discouraged from performing laparoscopic procedures due to lack of qualified residents to assist.^29^

Having a target number of laparoscopic cases defined by the local department or college was a highly rated factor in encouraging laparoscopic training in South Africa, and particularly important in an environment where barriers to laparoscopic training already exist.^16^

## Discussion

The value of laparoscopic training in resource-deprived LMIC regions is highly debated. Funding is a major barrier to training, restricting access to equipment and trainers, meaning that laparoscopy is usually limited to urban centres in more affluent LMICs. The small-scale nature of most of the training programmes means that sustainability and long-term cost-effectiveness of laparoscopic surgery need to be explored further. With increased need for access to surgical care, frugal technological solutions^36^ are required to reverse this trend in low-resource settings. Modified laparoscopic techniques, such as gasless laparoscopic surgery,^37^ could become an affordable solution and provide better access to surgical care over open surgical technique. However, dedicated training programmes are required to facilitate its formal adoption in rural settings.

Accessing and maintaining equipment has been shown to be consistently difficult, as demonstrated by the heavy reliance on donations across many training programmes. Experienced, local trainers are often scarce and they may not be willing or able to perform the role. Reliance on expatriate and foreign surgeons has not produced a reliable and sustainable training model. With the growing need to improve surgical access in low-resource LMIC regions, local trainers need to be encouraged and motivated to undertake laparoscopic training that is appropriate for the local context. To prevent widening inequalities in access to laparoscopic surgery, innovative solutions for training are required, including inexpensive immersive reality technologies.^17,36^ With increasingly reliable global internet coverage, tele-proctoring will likely offer a sustainable solution where lack of access to trainers in LMICs is problematic.

The implementation of laparoscopic training programmes requires collaboration between multiple stakeholders, which is often influenced by local socio-cultural factors. This can only be addressed through guidance and policy from professional and governmental bodies,^10^ incorporating laparoscopic surgery in all rural surgery training curricula.

Surgical department structure must be reorganised to accommodate greater training opportunities for residents, and hierarchical structures and traditional training methods need to be challenged.

### Limitations

Only 18 LMICs were specifically covered by this review. Many studies only investigated laparoscopic training in one centre, and training was often focused on the most rural and resource-deprived areas, which limits the generalisability of the findings. Access to surgical resources can vary widely within LMICs, so barriers to laparoscopy in one region may not reflect the whole country.

Inclusion of only English-language texts and exclusion of conference abstracts and non-peer-reviewed studies limited the scope of this review. There is a risk of publication and reporting bias, as published results are more likely to be from successful training programmes. Risk of bias of individual studies was not formally assessed due to the types of studies included. This review spans a period of 24 years, although the majority of studies are from the last decade, meaning that recent changes in healthcare provision might not have been captured.

## Supplemental Material

sj-pdf-1-tdo-10.1177_0049475521998186 - Supplemental material for Barriers to training in laparoscopic surgery in low- and middle-income countries: A systematic review Click here for additional data file. Supplemental material, sj-pdf-1-tdo-10.1177_0049475521998186 for Barriers to training in laparoscopic surgery in low- and middle-income countries: A systematic review by Ellen Wilkinson, Noel Aruparayil, J Gnanaraj, Julia Brown and David Jayne in Tropical Doctor

sj-pdf-2-tdo-10.1177_0049475521998186 - Supplemental material for Barriers to training in laparoscopic surgery in low- and middle-income countries: A systematic review Click here for additional data file. Supplemental material, sj-pdf-2-tdo-10.1177_0049475521998186 for Barriers to training in laparoscopic surgery in low- and middle-income countries: A systematic review by Ellen Wilkinson, Noel Aruparayil, J Gnanaraj, Julia Brown and David Jayne in Tropical Doctor
