# Supplementary material for: Barriers to training in laparoscopic surgery in low- and middle-income countries: A systematic review
Source: Trop Doct. 2021 Apr 13;51(3):408–14. doi: 10.1177/0049475521998186 (PMC8411480; doi:10.1177/0049475521998186)
Supplement: sj-pdf-1-tdo-10.1177_0049475521998186 - Supplemental material for Barriers to training in laparoscopic surgery in low- and middle-income countries: A systematic review [file sj-pdf-1-tdo-10.1177_0049475521998186.pdf]

Barriers to training in laparoscopic surgery in low- and middle- income countries: A systematic review

E Wilkinson, N Aruparayil, J Gnanaraj, J Brown, D Jayne

Corresponding Author: Mr Noel K Aruparayil, NIHR Clinical Research Fellow, Global Health Research Group - Surgical Technologies, Leeds Institute of Medical Research at St James's, University of Leeds, LS9 7FT United Kingdom. Email: n.k.aruparayil@leeds.ac.uk

Appendix 1: Search Strategy

Database: Ovid MEDLINE(R) and Epub Ahead of Print, In-Process & Other Non-Indexed Citations and Daily <1946 to April 03, 2020>
Date: April 06 2020

	Search	Results
1	Developing Countries/	(74077)
2	(Low* income* adj3 (countr* or nation* or economy or economies)).tw.	(7145)
3	(LIC* adj3 (countr* or nation* or economy or economies)).tw.	(1011)
4	((Developing or "under developed" or underdeveloped or less-developed or "less* developed" or "third world") adj3 (countr* or nation* or economy or economies)).tw.	(67138)
5	((Underserved or "under served" or deprived or poor*) adj3 (countr* or nation* or economy or economies)).tw.	(5531)
6	((Developing or "less* developed" or less-developed or "under developed" or underdeveloped) adj2 world).tw.	(8611)
7	"transition* countr*".tw.	(310)
8	Afghanistan/ or Benin/ or Burkina Faso/ or Burundi/ or Central African Republic/ or Chad/ or "Democratic Republic of the Congo"/ or Eritrea/ or Ethiopia/ or Gambia/ or Guinea/ or Guinea-Bissau/ or Haiti/ or "Democratic People's Republic of Korea"/ or Liberia/ or Madagascar/ or Malawi/ or Mali/ or Mozambique/ or Nepal/ or Niger/ or Rwanda/ or Sierra Leone/ or Somalia/ or South Sudan/ or Syria/ or Tajikistan/ or Tanzania/ or Togo/ or Uganda/ or Yemen/	(87338)
9	(Afghan* or Benin* or "Burkina Faso" or Burundi* or "Central African Republic" or Chad* or "Democratic Republic of the Congo" or Eritrea* or Ethiopia*).ab,in,ti.	(62107)
10	(Gambia* or Guinea* or Guinea-Bissau* or Haiti* or "North Korea*" or "DPR Korea*" or "Korea* DPR" or "Democratic people* republic of Korea*" or "Korea* democratic people* republic" or "DPRK").ab,in,ti.	(121311)
11	(Liberia* or Madagasca* or Malawi* or Mali* or Mozambique* or Nepal* or Niger or Rwanda*).ab,in,ti.	(661618)
12	("Sierra Leone" or Somalia* or "South Sudan*" or Syria* or "Syrian Arab Republic" or Tajikistan* or Tanzania* or Togo or Uganda* or Yemen*).ab,in,ti.	(56224)
13	1 or 2 or 3 or 4 or 5 or 6 or 7 or 8 or 9 or 10 or 11 or 12	(1018439)
14	(Low-middle income* adj3 (countr* or nation* or economy or economies)).tw.	(784)

15	(Lower-middle income* adj3 (countr* or nation* or economy or economies)).tw.	(737)
16	(LMIC* adj3 (countr* or nation* or economy or economies)).tw.	(3939)
17	LMIC*.tw.	(4271)
18	Angola/ or Bangladesh/ or Bhutan/ or Bolivia/ or Cabo Verde/ or Cambodia/ or Cameroon/ or Comoros/ or Congo/ or Cote d'Ivoire/ or Djibouti/ or Egypt/ or El Salvador/ or Ghana/ or Honduras/ or India/ or Indonesia/ or Kenya/ or Kyrgyzstan/ or Laos/ or Lesotho/ or Mauritania/ or Micronesia/ or Moldova/ or Mongolia/ or Morocco/ or Myanmar/ or Nicaragua/ or Nigeria/ or Pakistan/ or Papua New Guinea/ or Philippines/ or Atlantic Islands/ or Melanesia/ or Senegal/ or Sudan/ or Swaziland/ or Timor-Leste/ or Tunisia/ or Ukraine/ or Uzbekistan/ or Vanuatu/ or Vietnam/ or Zambia/ or Zimbabwe/	(305538)
19	(Angola* or Bangladesh* or Bhutan* or Bolivia* or "Cabo Verde" or Cambodia* or Cameroon* or Comoros* or Congo or "Cote D'Ivoire" or "Ivory Coast" or Djibouti* or Egypt* or "El Salvador" or Ghana* or Honduras*).ab,in,ti.	(158606)
20	(India* not "american indian*").ab,in,ti.	(638155)
21	(Indonesia* or Kenya* or Kiribati* or Kyrgyzstan* or "Kyrgyz Republic" or Lao or Laos or Lesotho or Mauritania* or Micronesia* or Moldova* or Mongolia* or Morocco* or Moroccan* or Myanmar* or Burma or Burmese).ab,in,ti.	(95077)
22	(Nicaragua* or Nigeria* or Pakistan* or "Papua New Guinea*" or Philippines or Filipino or "Sao Tome and Principe" or Senegal* or "Solomon Islands").ab,in,ti.	(131360)
23	((Sudan* not "South Sudan*") or Swaziland* or Eswatini* or Timor* or Tunisia* or Ukrain* or Uzbekistan* or Vanuatu* or Vietnam* or "West Bank" or Gaza or Zambia* or Zimbabw*).ab,in,ti.	(97795)
24	14 or 15 or 16 or 17 or 18 or 19 or 20 or 21 or 22 or 23	(1150931)
25	(Upper-middle income* adj3 (countr* or nation* or economy or economies)).tw.	(429)
26	(UMIC* adj3 (countr* or nation* or economy or economies)).tw.	(44)
27	(Middle income* adj3 (countr* or nation* or economy or economies)).tw.	(17379)
28	(Low adj2 middle income* adj3 (countr* or nation* or economy or economies)).tw.	(13778)
29	Albania/ or Algeria/ or American Samoa/ or Argentina/ or Armenia/ or Azerbaijan/ or "Republic of Belarus"/ or Belize/ or "Bosnia and Herzegovina"/ or Botswana/ or Brazil/ or Bulgaria/ or China/ or Colombia/ or Costa Rica/ or Cuba/ or Dominica/ or Dominican Republic/ or Equatorial Guinea/ or Ecuador/ or Fiji/ or Gabon/ or "Georgia (Republic)"/ or Grenada/ or Guatemala/ or Guyana/ or Iran/ or Iraq/ or Jamaica/ or Jordan/ or Kazakhstan/ or Kosovo/ or Lebanon/ or Libya/ or "Macedonia (Republic)"/ or Malaysia/ or Indian Ocean Islands/ or Mauritius/ or Mexico/ or Montenegro/ or Namibia/ or Paraguay/ or Peru/ or Romania/ or Russia/ or Samoa/ or "Independent State of Samoa"/ or Serbia/ or Sri Lanka/ or South Africa/ or Saint Lucia/ or "Saint Vincent and the Grenadines"/ or Suriname/ or Thailand/ or Tonga/ or Turkey/ or Turkmenistan/ or Venezuela/	(589289)

30	(Albania* or Algeria* or "American Samoa*" or Argentina* or Armenia* or Azerbaijan* or Belarus* or Belize* or Bosnia* or "Bosnia adj2 Herzegovina*" or Botswana* or Brazil* or Bulgaria* or China* or Chinese or Columbia* or "Costa Rica*" or Cuba* or Dominica* or "Dominican Republic" or "Equatorial Guinea*").ab,in,ti.	(2481634)
31	(Ecuador* or Fiji* or Gabon* or Georgia* or Grenada* or Guatemala* or Guyana* or Guyanese or Iran* or Persia* or Iraq* or Jamaica* or Jordan* or Kazakhstan* or Kosovo* or Lebanon* or Lebanese or Libya* or Macedonia* or Malaysia* or Maldives or "Marshall Islands" or Mauritius* or Mauritian or Mexic*).ab,in,ti.	(635123)
32	(Montenegro* or Namibia* or Nauru* or Paraguay* or Peru* or Romania* or Russia* or Samoa* or Serbia* or "South Africa*" or "Sri Lanka*" or "St Lucia*" or "Saint Lucia*" or "St Vincent adj3 Grenadines" or "Saint Vincent adj3 Grenadines").ab,in,ti.	(361674)
33	(Suriname* or Thai* or Tonga* or Turkey* or Turkish or Turkmenistan* or Tuvalu* or Venezuela*).ab,in,ti.	(360109)
34	25 or 26 or 27 or 28 or 29 or 30 or 31 or 32 or 33	(3852052)
35	13 or 24 or 34	(5619483)
36	(laparoscop* or "minimal* invasive").tw.	(179087)
37	exp Laparoscopy/	(96617)
38	Minimally Invasive Surgical Procedures/	(25180)
39	36 or 37 or 38	(201002)
40	((train* or educat* or teach* or pract* or program*) adj7 (laparoscop* or "minimal* invasive")).tw.	(5717)
41	exp education, professional/ or exp teaching/	(334977)
42	40 or 41	(339036)
43	35 and 39 and 42	(1276)
44	limit 43 to english language	(1141)
