# Supplementary material for: Barriers to training in laparoscopic surgery in low- and middle-income countries: A systematic review
Source: Trop Doct. 2021 Apr 13;51(3):408–14. doi: 10.1177/0049475521998186 (PMC8411480; doi:10.1177/0049475521998186)
Supplement: sj-pdf-2-tdo-10.1177_0049475521998186 - Supplemental material for Barriers to training in laparoscopic surgery in low- and middle-income countries: A systematic review [file sj-pdf-2-tdo-10.1177_0049475521998186.pdf]

Barriers to training in laparoscopic surgery in low- and middle- income countries: A systematic review

E Wilkinson, N Aruparayil, J Gnanaraj, J Brown, D Jayne

Corresponding Author: Mr Noel K Aruparayil, NIHR Clinical Research Fellow, Global Health Research Group - Surgical Technologies, Leeds Institute of Medical Research at St James's, University of Leeds, LS9 7FT United Kingdom. Email: n.k.aruparayil@leeds.ac.uk

Appendix 2: Inclusion and Exclusion Criteria

**Inclusion Criteria**	**Exclusion Criteria**
English language papers	
Full-text accessible through authors' subscriptions	
Papers published in a peer-reviewed journal	
Journal articles or reviews	Conference abstracts/letters/meeting abstracts/case reports/editorials
Papers about training in LMICs	Papers about training in high-income countries (HICs)
Address barriers to laparoscopic training	
Training of qualified health professionals	Training of medical students
Pertaining specifically to abdominal laparoscopy (defined for this paper to include gastrointestinal, hepatobiliary	Pertaining specifically to non-abdominal laparoscopy e.g. Neurology, urology, nephrology

and obstetric/gynaecological) or laparoscopy in general	
	Advanced laparoscopy such as robotics or transplant surgery
	Paediatric surgery
